# Pulsed photothermal interferometry for spectroscopic gas detection with hollow-core optical fibre

**DOI:** 10.1038/srep39410

**Published:** 2016-12-23

**Authors:** Yuechuan Lin, Wei Jin, Fan Yang, Jun Ma, Chao Wang, Hoi Lut Ho, Yang Liu

**Affiliations:** 1 Photonic Sensors Research Laboratory, Department of Electrical Engineering, The Hong Kong Polytechnic University, Hong Kong, China; 2 Photonic Sensors Research Laboratory, The Hong Kong Polytechnic University Shenzhen Research Institute, Shenzhen, China; 3 Department of Mechanical Engineering, The Hong Kong Polytechnic University, Hong Kong, China

## Abstract

Gas detection with hollow-core photonic bandgap fibre (HC-PBF) and pulsed photothermal (PT) interferometry spectroscopy are studied theoretically and experimentally. A theoretical model is developed and used to compute the gas-absorption-induced temperature and phase modulation in a HC-PBF filled with low-concentration of *C*_2_*H*_2_ in nitrogen. The PT phase modulation dynamics for different pulse duration, peak power and energy of pump beam are numerically modelled, which are supported by the experimental results obtained around the P(9) absorption line of *C*_2_*H*_2_ at 1530.371 nm. Thermal conduction is identified as the main process responsible for the phase modulation dynamics. For a constant peak pump power level, the phase modulation is found to increase with pulse duration up to ~1.2 μs, while it increases with decreasing pulse duration for a constant pulse energy. It is theoretically possible to achieve ppb level detection of *C*_2_*H*_2_ with ~1 m length HC-PBF and a pump beam with ~10 ns pulse duration and ~100 nJ pulse energy.

Trace gas detection is important for a range of applications such as environmental and safety monitoring as well as medical breath analysis. Compared with traditional electrochemical and semiconductor sensors, optical fibre sensors based on laser absorption spectroscopy offer good selectivity and sensitivity, with additional advantages such as capability for remote detection and immunity to electromagnetic interference. Earlier optical fibre gas sensors used micro-optic open-path cells and the sensitivity is limited due to the weak absorption of gases within the transmission windows of the standard (silica) optical fibres and the difficulty in building long path-length absorption cells with compact size^1^. The use of HC-PBF allows the construction of compact absorption gas cells with optical path length of many meters^2^,^3^. However, the performance of these gas sensors was found limited by mode interference noise^4^,^5^. So far the best result reported with direct absorption optical fibre gas sensors is ~1 ppm (acetylene) with 13-m-long sensing HC-PBF^5^.

Photothermal interferometry (PTI) is an ultra-sensitive spectroscopic technique for gas-phase material analysis^6^,^7^,^8^. Instead of measuring optical spectral attenuations, PTI detects the absorption-induced photothermal (PT) phase modulation via optical interferometry. A pump-probe configuration is typically used, in which absorption of the pump beam generates localized heating, modulates the refractive index (RI) and the phase of the probe beam traveling through the material. PTI has been implemented with free-space optics, operating at primarily infrared wavelengths where absorptions of many gas molecules are the strongest. However, due to optical alignment loss, cost and complexity issues, the light-matter interaction length of free-space systems is limited to several tens of centimeters^7^, making them less effective at near infrared wavelengths where gas absorptions are relatively weak. Recently we studied PTI with a HC-PBF and demonstrated detection of *C*_2_*H*_2_ down to ppb level with a dynamic range of six orders of magnitude, these results are nearly three orders of magnitude better than previously reported gas sensors using HC-PBF^6^. The use of hollow-core fibres as the sensing platform offers several distinct advantages over free-space approaches. A HC-PBF confines light and gas sample simultaneously within the hollow-core, offering nearly 100% overlap between the sample and light. The PT phase modulation is proportional to pump light intensity instead of power, and for the same pump power level, a HC-PBF would offer a much higher light intensity due to its much smaller mode field diameter compared with the free-space approaches. Ultra-sensitivity could be achieved by use of a longer length of fibre while it maintains the compactness since a HC-PBF can be coiled down to centimeters without obvious increase in transmission loss^2^.

In our first demonstration of PTI with hollow-core optical fibre^6^, a continuous wave laser with its wavelength/intensity modulated at 50 kHz was used as the pump source. The PT phase modulation was detected by an all-fibre Mach-Zehnder interferometer (MZI) and lock-in detection was used to improve the signal to noise ratio (SNR). The signal level was found to increase linearly with the pump power level. Since most of the high power lasers currently available operate in a pulsed mode, the use of these pulsed lasers would enable higher signal level and hence higher detection sensitivity. However, no work on PT effect in a HC-PBF with a pulsed laser source has been reported so far to our knowledge.

In this paper, we report the results of our theoretical and experimental investigation of the dynamics of PT phase modulation in a gas-filled hollow-core optical fibre with a pulsed pump laser source. First of all, we present a theoretical model and formulations for computing the PT phase modulation, as well as the numerical results obtained with a particular HC-PBF. Then we presents the experimental setup and results obtained with *C*_2_*H*_2_ (with *N*_2_ as the buffer gas) for different durations and peak power levels of pump pulses, as well as the measurement results of sensitivity and line-shape with a low peak power pulsed pump laser. Finally, discussions and conclusions are drawn and guidelines for future work on pulsed PTI with HC-PBF are presented.

## Results

### A model for computing PT phase modulation

**Figure 1:**
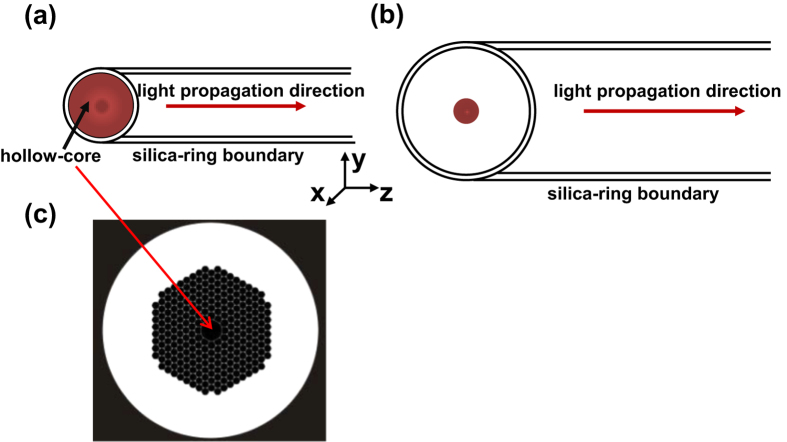
Model for computing PT phase modulation. (**a**) A HC-PBF and (**b**) A free-space beam within a cylindrical tube filled with gas. The red region indicates the area of the light beam. The region inside the ring is filled with gas to be measured. In the model for the HC-PBF, the beam diameter is similar to that of the ring, while it is much smaller than the ring for the free-space open-path system. (**c**) Schematic fibre cross section of the HC-1550-02 fibre from NKT Photonics data sheet.

The PT phase modulation in a gas-filled HC-PBF may be studied by use of a simplified model shown in Fig. 1a. The model includes three regions: an inner circular region with the diameter of the hollow-core which is filled with the gas sample to be measured, a silica ring region with a thickness equal to the wall-thickness of the hollow-core, and an outer gas region filled with the same gas. In this model, the micro-structured cladding of the HC-PBF is approximated by an outer gas region because of the very large gas-filling ratio (>90%^2^). An optical pump beam propagating in the *z*-direction is absorbed by the gas molecules and acts as the heat source, which modulates the refractive index of gas samples in the hollow-core fibres and hence the phase of the probe beam travelling in the same fibre. The same model may be used to study the PT phase modulation for a collimated free-space beam inside a cylindrical gas chamber, and in this case, the diameter of the cylindrical tube can be much larger than that of the pump beam, as shown in Fig. 1b. The cross section of a typical HC-PBF is shown in Fig. 1c.

Even with the simplified model shown in Fig. 1, an accurate analysis of the PT modulation dynamics would be a very complex process. For pump pulse duration from a few ns to a few *μ*s, which is the case for our experimental investigation, some assumptions/approximations may be made to simplify the analysis.

First, the time for gas molecules to be excited from ground state to higher energy state is fast enough to be ignored since this process is usually in the time scale of picoseconds.

Second, we only consider *V* → *R, T* relaxation process. For most gases under standard temperature and pressure (s.t.p) condition, other relaxation processes are typically much faster than the *V* → *R, T* process^8^. For gas sample studied here (i.e., *C*_2_*H*_2_ with *N*_2_ as buffer gas), under s.t.p condition, the *V* → *R, T* relaxation time *τ* is ~74 ns^9^.

**Figure 2:**
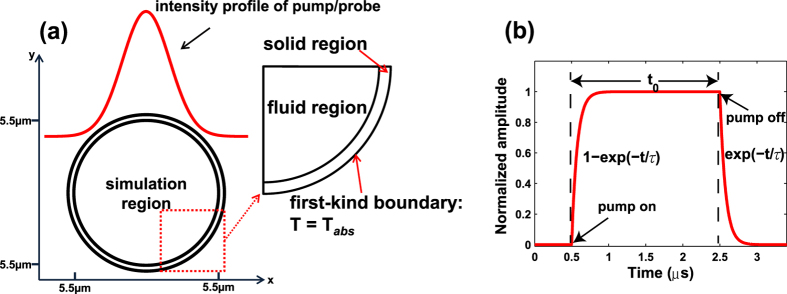
Details of the model. (**a**) The region within the circular ring encloses the gas sample and is the domain within which our simulation is conducted. The intensity profiles (the fundamental mode) for both the pump and probe are Gaussian and in our simulation they are assumed to have the same mode field radius. However, the model can also be used for pump and probe beams that have different mode field parameters. The temperature at the outer ring boundary is regarded as constant and continuous temperature change is assumed between solid and fluid regions. (**b**) Shape of a typical heating pulse S(*t*) used in our simulation. For this particular pulse, the pump pulse duration is *t*_0_ = 2*μs* and the relaxation time *τ* is 74 ns.

Third, we assume that the absorption is weak, and the pump intensity and hence the heat generated is invariant along the direction of propagation. This allows us to use the simplified 2D models shown in Fig. 2. It can easily be extended to the case of stronger absorption by dividing the optical path into small sections and, within each section the pump intensity may be regarded as invariant.

Fourth, the thermal dynamics process in the hollow-core may be approximately regarded the same as that in the continuum regime. Under atmospheric pressure, the Knudsen number (*Kn* = Λ/*d*, where Λ is the mean free path of gas molecules and *d* is the geometry diameter of gas chamber) is in the orders of 10^−6^ for *N*_2_ gas (the concentration of *C*_2_*H*_2_ is much smaller) in a 1-mm-diameter tube. For a HC-PBF with hollow-core diameter of *d* = 11 *μm*, the Knudsen number is about 0.008 for *N*_2_ gas. It has been shown that with *Kn* from 0.001 and 0.1, the continuum regime assumption is still valid within most of the fluid region while in the region adjacent to the boundary between fluid and solid, the slip flow condition should be considered for a more accurate model^10^. However, as the fundamental mode profile of the pump (and probe) beam in a HC-PBF is approximately of a Gaussian shape (as illustrated in Fig. 2a), and the light intensity near the core/cladding boundary region is significantly lower than that at the core center. It means that the temperature change near the boundary is much smaller than that near the center, and its effect on the phase modulation of probe beam should be very small. Hence, the exact flow condition near the boundary would have very little effect on the overall phase modulation and may not need to consider for purpose of calculating phase modulation.

Fifth, the thermal conduction is regarded as the dominant heat dissipation process. Under the condition of the same or similar beam radius (*w*_*p*_) for the pump and probe beams and ignoring the mutual influence between conduction and convection processes, the natural convection velocity around laser radiation region may be estimated by^11^:


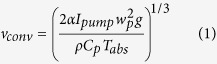


where *α(cm*^−1^) = *α*_0_*C*_0_. *α*_0_(*cm*^−1^) is the peak absorption coefficient of the absorptive gas for a relative concentration of 100% at s.t.p, which equals to 1.165 *cm*^−1^ for the P(9) absorption line in *v*_1_ + *v*_3_ overtone band of *C*_2_*H*_2_. *C*_0_ is a relative gas concentration. *I*_*pump*_ is pump laser intensity, *g*(=9.8 *m/s*^2^) is the acceleration of gravity, *ρ*(=1.165 *kg/m*^3^) is *N*_2_ gas density, *C*_*p*_(=1040 *J*/(*kg* · *K*)) is the specific heat of *N*_2_ gas molecules and *T*_*abs*_(=293.15 *K*) is the ambient temperature.

The thermal conduction velocity may be estimated by^7^:


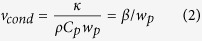


where *κ*(=0.0256 *W*/ (*m* · *K*)) is the thermal conductivity of *N*_2_ buffer gas, *β* = *κ/ρC*_*p*_ ≈ 2.0 × 10^−5^ *m*^2^/*s* is the thermal diffusivity of the buffer gas. Under s.t.p condition, in a HC-PBF with beam radius of ~4.03 *μm* 12, the natural convection velocity is estimated to be ~1 × 10^−4^ *m/s* with a peak pump power level of 25 mW and 100 ppm (parts per million by volume) *C*_2_*H*_2_ gas while the thermal conduction velocity is 5 *m/s*. This would give a thermal conduction time of the order of *μ*s and a convection time of the order of ms. For the pump pulse duration from ns to *μ*s, the natural convection process would not be fast enough to catch up with the PT signal change. Therefore, within our observation time, the natural convection process could be ignored.

Based on the assumption above, the temperature distribution within the hollow-core may be obtained by solving the heat transfer equation^7^:


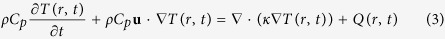


where **u** is the velocity field with an initial value of zero and *Q(r, t*) (*W/m*^3^) is the volume heat source.

The absorption of the pump beam results in heating around the center of the HC-PBF, and the volume heat source *Q(r, t*) may be regarded as invariant in *z*-direction under the condition of weak absorption and expressed in the form of:


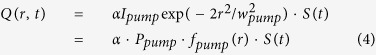


where 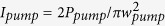 is the peak intensity of pump and the pump beam is assumed to have a Gaussian profile with a beam radius (peak power down to 1/*e*^2^ of its maximum) of *w*_*pump*_and peak power of *P*_*pump*_. S(*t*) is the pulse waveform in the time domain, and *f*_*pump*_(*r*) is the area-normalized intensity profile and takes the form of^13^:


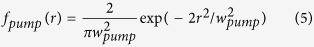


For small temperature changes, the temperature at the outer boundary of the silica ring may be regarded as constant and equal to the ambient, which is the so-called first-kind boundary condition^13^. The temperature distribution in the inner circular gas region and the silica ring region is regarded as varying continuously, and all the thermal properties of gas material are also regarded as constant considering the very small temperature changes. With these conditions, Equation (3) may be solved numerically and temperature changes Δ*T(r, t*)(=*T(r, t*) − *T*_*abs*_) could be obtained.

The refractive index changes in the gas-filled hollow-core region may then be obtained through the Lorentz-Lorentz relation^7^:


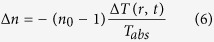


Under the assumption of constant pressure, the refractive index changes could also be expressed in terms of density changes, which have a similar expression as that in terms of temperature changes^7^,^13^.

The change of effective refractive index of a propagating probe mode may be calculated by performing the following integration over the cross section of hollow-core (the fluid region) by using^14^:


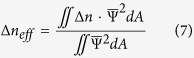


where 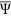 is the electrical field profile of probe beam in the HC-PBF. The fundamental mode of the probe beam is also approximately Gaussian and may be expressed as:


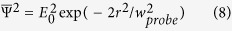


where*E*_0_is the electrical field strength and the integration of the electrical field satisfies 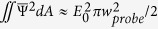.

The overall phase changes of the probe beam, due to PT effect, may be calculated by using:


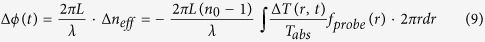


where *n*_0_ is the refractive index of gas sample under s.t.p condition, for nitrogen *n*_0_ ~ 1 + 2 × 10^−4^. *L* is the length of HC-PBF (in our simulation and experiment *L* = 0.62 m), *λ* is the wavelength of probe beam. *f*_*probe*_(*r*) is the area-normalized intensity profile of probe beam, which has the same expression as Equation (5) but with *w*_*pump*_ replaced by *w*_*probe*_. For the cases studied here, the probe beam has approximately the same profile as the pump beam, and hence we may use *w*_*pump*_ = *w*_*probe*_ = *w*_*p*_.

### Computed results of PT phase modulation

The pulse waveform function S(*t*) used in our simulation is illustrated in the Fig. 2b. After the pump pulse is turned on, the heat rises exponentially to a constant level by following the function of 1 − exp(−*t/τ*), where *τ* is the thermal relaxation time of excited gas molecules. On the other hand, when the pump pulse is turned-off, the heat follows an exponential decay exp(−*t/τ*)^7^.

**Figure 3:**
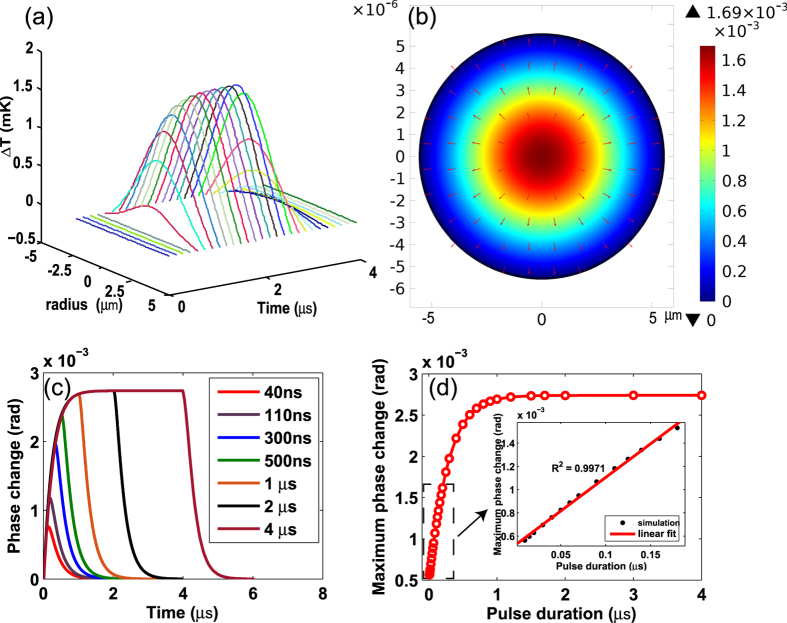
Computation results. The HC-PBF has a core radius of 5.5 *μm*, the beam radius of pump beam is 4.03 *μm* and the duration of the pump pulse is 2 *μs*. The peak power of the pump is 25 mW. The HC-PBF is 0.62-m-long and filled with 100 ppm *C*_2_*H*_2_ in nitrogen. (**a**) Time evolution of temperature distribution Δ*T(r, t*); (**b**) 2D plot of Δ*T(r, t*) at the time of 1.5 *μs* after the pump pulse is turned on, with arrows indicating the direction of heat flow. The unit of temperature in the plot is Kelvin. (**c**) Computed PT phase modulation in a HC-PBF for different pulse durations. (**d**) Maximum phase change as function of pulse duration of the pump.

Figure 3a shows the calculated time evolution of temperature rise Δ*T(r, t*) in a HC-PBF containing 100 ppm of *C*_2_*H*_2_. The pump pulse has duration of 2 *μs* and a peak power of 25 mW, and its wavelength is tuned to the P(9) absorption line of *C*_2_*H*_2_ at 1530.371 nm. The maximum Δ*T(r, t*) occurs at the center of the fibre and is less than 0.002 K. Figure 3b shows the 2D plot of Δ*T(r, t*) at the time of 1.5 *μs* after the pump pulse is turned on. The arrows in Fig. 3b represent the direction of heat flux, indicating that the heat tends to dissipate outward to reach thermal equilibrium.

Figure 3c shows the computed phase modulation dynamics for different pulse durations from 40 ns to 4 *μs*, and the peak phase change as function of pulse duration for constant peak pump power is plotted in Fig. 3d. The peak phase change increases approximately linearly with the increasing pulse duration till about ~200 ns. This value is quite close to the thermal conduction time to a quarter of the beam radius 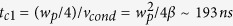 similar to the results of free-space system^7^. When the pulse duration is beyond ~1.2 *μs*, the peak phase modulation does not increase significantly with increasing pulse duration, indicating an optimal pulse duration of ~1.2 *μs* to achieve maximum peak phase modulation for a constant peak pump power. This pulse duration coincides approximately with the thermal conduction time to the hollow-core boundary, which is estimated to be *t*_*c*2_ = *a/v*_*cond*_ ~ 1.1 *μs*, where *a* is the radius of hollow core. This shows that gas thermal conduction plays a major role in the phase modulation dynamics in the HC-PBF.

**Figure 4:**
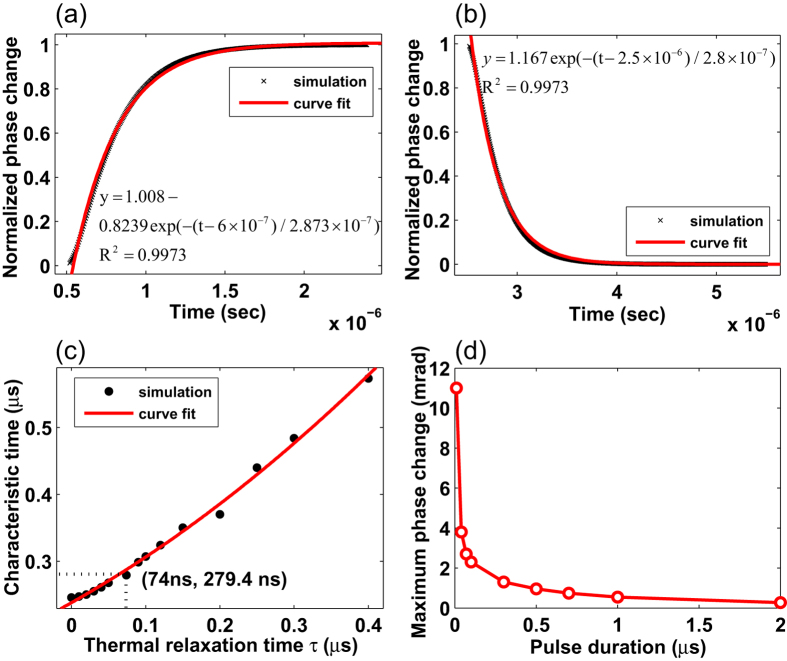
Computation results. (**a**) Leading and (**b**) Trailing part of PT phase modulation with pulse duration of 2 *μs* together with curve fittings. The amplitude of phase changes has been normalized. (**c**) The relationship between characteristic time constant and thermal relaxation time. (**d**) Peak phase modulation with different pump pulse duration but constant pulse energy of 5 *nJ*. The simulation is conducted with 0.62-m-long HC-PBF filled with 100 ppm*C*_2_*H*_2_.

By fitting the leading and trailing edges of the phase modulation signal for the 2 *μs* duration of pump pulse (Fig. 4a,b), we obtained a time constant of 287 ns and 280 ns respectively which represents the characteristic time that the PT phase change will rise up to 1 − 1/*e* or decay down to 1/*e* of its maximum value. These values are about one quarter the value of *t*_*c*2_ (i.e., *t*_*c*2_/4 ~ 275*ns*), showing that the thermal conduction is the main process that determines the rise and falling behavior of the phase modulation.

We also examined the role of thermal relaxation time *τ* on characteristic time constant of leading/trailing edge of the phase modulation signals. By varying *τ* from 5 ns to 400 ns while keeping other parameters unchanged, and fitting the trailing edge, the characteristic time constant of exponential curve was found and shown in Fig. 4c. The time constant increases from 240 ns to 550 ns. For our case *τ* = 74 *ns*, the characteristic time is about 280 ns, the conduction process is dominant, but for other gases with *τ* ≫ *t*_*c*2_/4, the *τ* could induce considerable the curve broadening. For *τ* ≫ *t*_*c*2_, this effect on the optimal pulse duration will be significant and required further investigations.

The results obtained for pulsed pump modulation would be a useful indication of the frequency dependent phase modulation for the sinusoidally modulated continuous-wave pumping scheme. We would expect that the PT phase modulation is frequency-independent at low modulation frequencies and started to decrease when the modulation frequency is increased beyond a critical frequency at 1/2*t*_*c*2_, which is ~450 kHz for the HC-PBF used here. For higher modulation frequency beyond ~1/2*t*_*c*1_, which is about 2 MHz, the phase modulation is expected to be approximately inversely proportional to the modulation frequency. Further experiments are currently on-going to verify such dependence.

For the 25 mW peak pump power, 100 ppm *C*_2_*H*_2_ gas concentration and 0.62-m-long HC-PBF, the PT maximum peak phase modulation is computed to be 0.002739 rad with the pump wavelength tuned to the P(9) line of *C*_2_*H*_2_ and a probe wavelength of 1553 nm. Since for fixed pulse duration, the maximum phase modulation is expected to increase linearly with the peak pump power, we may then obtain a normalized phase modulation coefficient: in terms of gas concentration 1.767 × 10^−6^ *rad* · *ppm*^−1^ · *mW*^−1^ · *m*^−1^ or 1.517 *rad* · *cm* · *mW*^−1^ · *m*^−1^ in terms of absorption coefficient.

The discussion above is for constant peak pump power with different pump pulse durations. If we now assume a constant pulse energy of 5 *nJ*, the maximum PT phase modulation as function of pulse duration is calculated and plotted in Fig. 4d. In obtaining the results, we considered an independent single pulse, i.e., the pulse repetition is sufficient slow that the two subsequent PTI pulse signals would not overlap with each other. Obviously, narrower pulse duration would result in a larger phase modulation. For a pulse duration of 10 ns, the maximum phase change for a 5 *nJ* pulse can be as high as 0.011 rad for 0.62 m-long HC-PBF, 100 ppm gas concentration. The normalized phase modulation coefficient may then be determined as 0.03548 *rad* · *ppm*^−1^ · *μJ*^−1^ · *m*^−1^. With a higher energy of 100 *nJ*, the achievable phase modulation would be ~0.3 rad using 100 ppm gas sample and 1-m-long HC-PBF. With a demodulation system with minimum detectable phase change of 1 *μrad*, the minimum detectable *C*_2_*H*_2_ concentration would be about 1 ppb. However, if the pulse energy is too high (e.g., at the order of *μJ* to *mJ* with pulse duration of nano-seconds), the temperature rise would be large that the first-boundary condition would not be valid any more. Further work is needed to study the phase modulation with higher energy pulses.

From equation (4), it can be seen that for a constant peak power, the peak value of *Q(r, t*) is inversely proportional to the spot size of the pump and hence we may expect that larger phase modulation would be achieved for a smaller beam area. However, the probe phase modulation is also affected by the dynamics of thermal conduction, which depends on several factors such as the core size of the hollow tube that contains the gas sample. Keeping the spot size of pump and probe beams un-changed as well as using the optimal pulse duration of *a/v*_*cond*_, the peak phase change for different core size was computed and is presented in the Table 1 (with 0.62 m-long HC-PBF, 25 mW peak power, 100 ppm acetylene in N_2_). The maximum PT phase modulation increases with the diameter of hollow-tube, indicating that the boundary has significant influence on the phase modulation, and the results obtained with infinite boundary, corresponding to the case of free-space approaches, are no longer valid for the HC-PBF. The reduced phase modulation for smaller core-size of hollow-tube may be explained by the better thermal conductivity of the tube material (silica here) as compared with the gas sample. The heat generated begins to dissipate before it reaches its maximum value. The smaller core size would result in faster thermal conduction which limits the maximum PT phase modulation.

As discussed above, the calculation method used in free-space^15^ may not be suitable for HC-PBF. For constant spot size of the pump and probe beams, larger core size would result in larger PT phase modulation under the condition of constant pump intensity. However, in real optical waveguides, larger core size would always mean larger pump spot size, which would reduce the pump intensity for constant pump power and hence PT phase modulation efficiency. Therefore, an optimal HC-PBF with appropriate core size should exist to achieve maximum phase modulation.

### Experimental results

**Figure 5:**
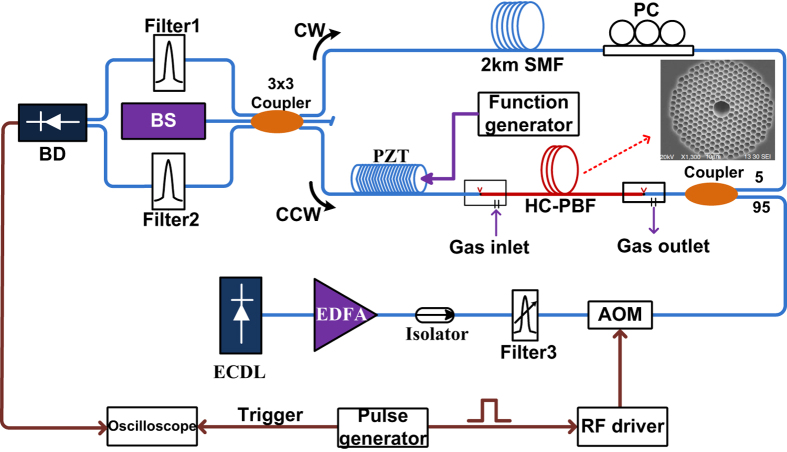
Experimental set-up for gas detection with 0.62-m-long HC-PBF. An external-cavity diode laser (ECDL) with wavelength around 1530.371 nm is used as pump beam. The tunable filter-3 filters out the noise accompanying the EDFA. The fixed wavelength filters (filter-1 and filter-2, both have center wavelength of 1553.33 nm and 3-dB bandwidth of 1 nm) are used to filter out the residual pump. AOM: acoustic-optic modulator. BS: broadband source (probe). PC: polarization controller. BD: balanced-detector. The output of BD is connected to an oscilloscope to observe the dynamics of the photothermal signal. A piezoelectric-transducer (PZT) is used for phase calibration purpose. The inset figure is the cross-sectional image of the sensing fibre (NKT Photonics’ HC-1550-02 fibre) that has a core diameter of ~11 *μm*.

The experimental set-up for studying the PT modulation dynamics is shown in Fig. 5. An external-cavity laser (ECDL) is utilized as a pump source and after passing through an erbium-doped amplifier (EDFA), it is modulated in intensity by an acoustic-optic modulator (AOM). The repetition rate of pulse modulation is set to 500 Hz. The sensing HC-PBF is 0.62-m long HC-1550-02 fibre (NKT Photonics) filled with 7500 ppm *C*_2_*H*_2_ in nitrogen. The nominal wavelength of the ECDL is calibrated by a standard *C*_2_*H*_2_ gas cell and tuned to the P (9) absorption line of *C*_2_*H*_2_ at 1530.371 nm.

The PT phase modulation in the HC-PBF is detected by the use of an all-fibre Sagnac interferometer with a 3 × 3 loop coupler. The output ports of the 3 × 3 coupler present a fixed phase difference of 2*π*/3 and the use of the balanced detection (BD) scheme gives a system output that is approximately linearly proportional to the phase modulation^16^. A super-luminescent light emitting diode (SLED) with bandwidth of 41 nm centered at 1545 nm is used as a broadband probe light source. After the 3 × 3 coupler, the divided probe beams travel through the same fibre loop but along opposite directions: clock-wise wave (CW) and counter-clock-wise (CCW) directions. In additional to the sensing HC-PBF, the Sagnac loop also includes a 2-km-long single mode fibre (SMF) as an optical delay line, which causes the CW and CCW waves travel through the sensing HC-PBF with a time difference of *t*_*d*_ ~ 10 *μs*.

Previously, we have used a fibre MZI for PT phase detection but active servo-control was required to stabilize the interferometer at quadrature and, in addition, the optical path lengths of the two interferometer arms need to be matched carefully to maintain long time stability^6^. The Sagnac configuration used here achieves linear phase to intensity conversion passively, avoiding the need of servo-control. It would also have better stability over the MZI since the CW and CCW beams propagate in the same fibre (opposite directions) and hence environmental disturbance would have less effect on the phase difference between the two interfering beams.

As discussed in Supplementary materials, the output waveform of Sagnac interferometer depends on pump pulse duration *t*_0_ and the loop delay time *t*_*d*_. For *t*_0_ < *t*_*d*_, the system output appears to be two identically shaped pulses but with reverse signs. Here in our experiments, we focus on the case that the pump duration is considerably shorter than the loop delay time *t*_*d*_ (here *t*_*d*_ ~ 10 *μs* corresponding to 2-km SMF); this ensures that the dynamics of PT phase modulation due to a single pump pulse could be observed clearly and directly from the output waveform.

**Figure 6:**
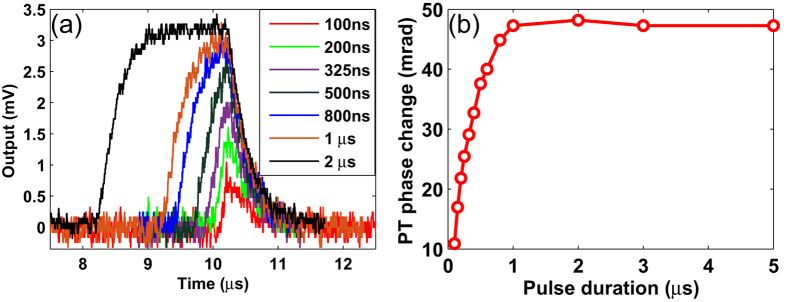
Experimental results. (**a**) Output from balanced detector (BD) for different pulse durations of the pump. (**b**) The peak phase change as function of pump pulse duration for a constant peak pump power of 20.2 mW delivered to the input end of the 0.62-m-long HC-PCF filled with 7500 ppm of *C*_2_*H*_2_ in nitrogen.

Figure 6a shows the output waveforms from BD for pump pulse duration from 100 ns to 2 *μ*s. The HC-PBF is 0.62 m long and filled with 7500 ppm *C*_2_*H*_2_ in nitrogen and the peak pump power delivered to the HC-PBF is 20.2 mW. The output signal closely resembles that predicted by the numerical model and the signal amplitude increases with pulse duration up to ~1.2 *μ*s and become flat for longer pulse duration, agreeing well with the numerical results in Fig. 3.

### Comparing experimental results with the modeling results

The output from BD may be converted to phase change of the probe in the HC-PBF by the following calibration process: an optical fibre phase modulator made by wrapping a piece of SMF on a piezoelectric-transducer (PZT) is connected into the Sagnac loop. A sinusoidal voltage of 50 kHz is used to drive the PZT to generate a *π* (amplitude) phase difference between the CW and CCW waves, which can be directly observed at the BD output by using an oscilloscope^17^. Assuming a linear relationship between the phase modulation and the PZT driving voltage, 0.5* rad* (observed peak amplitude) phase difference is obtained by adjusting the voltage applied to the PZT modulator, and the corresponding peak voltage of the BD output is measured to be 33* mV* (amplitude). Since for sinusoidal phase modulation, the amplitude of the phase difference is twice that of the CW (or CCW) wave^16^, the relationship between the peak voltage of the BD output and the amplitude of the CW (or CCW) phase modulation should be 33 *mV*/0.25* rad* = 132 *mV/rad*. However, for our pulsed phase modulation considered here, the amplitude of the phase difference is the same (without the factor of 2) as the phase modulation of CW (or CCW) wave. Hence the response of our Sagnac system to the pulsed modulation may be determined to be 66 *mV/rad*. The peak PT phase modulation corresponding to the peak output voltage shown in Fig. 6a is then determined to be 3.102 *mV*/66 *mV* × 1 *rad* = 0.047 *rad*.

With this voltage to phase conversation constant, the maximum phase change as function of the pump pulse duration can be calculated and shown in Fig. 6b. The maximum phase modulation increases with the pulse duration for up to 1.2 *μ*s, and remains more or less constant for pulse duration beyond this value. This agrees with the numerical prediction and discussion in Fig. 3.

Although 20.2 mW pump power is delivered to the input end of the HC-PBF, the power level in the HC-PBF may not be regarded as constant due to the absorption of the pump beam by *C*_2_*H*_2_. For the gas concentration of *C*_0_ = 7500 ppm, the weak absorption condition is no longer strictly valid and the average calibrated pump power over the length of 0.62 m is estimated as pump power 20.2 × (1 − exp(−*αL*)), i.e. ~8.4 mW. The computed maximum phase change for 2 *μ*s pulse is 0.069* rad* and there is about ~38% larger than the experimental value of 0.047 *rad*. The deviation between the theory and the experiment may be due to (i) phase calibration errors, (ii) mass diffusion and some other complicated processes that are ignored in our simplified model, (iii) the errors in determining the exact spot and core sizes of the HC-PBF that may vary slightly for different section of the HC-PBF, and (iv) the non-ideal heat yield–we have assumed that the light energy absorbed by gas molecules has been completely converted into heat via thermal relaxation process^8^, there would be other processes that could result in a less than 100% heat yield but are not considered here.

**Figure 7:**
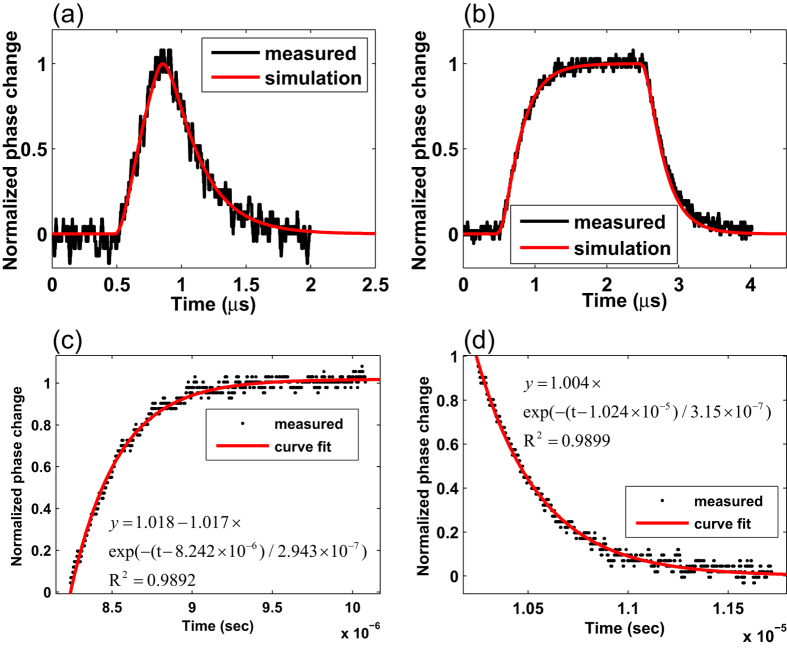
Comparison between experimental results and computation results. The experimental and simulation results of PTI signal with pump pulse duration of (**a**) 325 ns and (**b**)2 *μs*. (**c**) (**d**) The curve fitting of leading and trailing edges of PTI signal obtained with pump pulse duration of 2 *μs*.

To compare theory with experiment, the phase changes obtained experimentally are plotted in the same graph for two pump pulse durations of 325 ns and 2 *μ*s, as shown in Fig. 7a,b. The simulation is based on the basic assumption described earlier with a thermal relaxation time *τ* of 74 ns. The phase change has been normalized against its maximum value of the pulse to precisely observe the behaviors of leading and trailing edges of signals. The results between experiment and simulation are well matched, indicating that our model is sufficient in predicting the PT phase modulation dynamics in the *C*_2_*H*_2_-filled HC-PBF.

We also examined more closely the dynamics of the PT-induced phase modulation by fitting the leading and trailing edges of the PT signal with the 2 *μs* pump pulse to an exponential function and the results are shown in Fig. 7c,d. The characteristic time constants for leading and trailing edges are respectively 294 ns and 315 ns, respectively, close to the calculated characteristic time constant *t*_*c*2_/4 ~ 275 ns and also the computed results ~280 ns. These values may be regarded as close to the numerical results shown in Fig. 4, taking into account the curve fitting errors due to the considerable fluctuation of the experimental signals as shown in Fig. 7.

### Measurements of gas detection sensitivity and absorption lineshape

**Figure 8:**
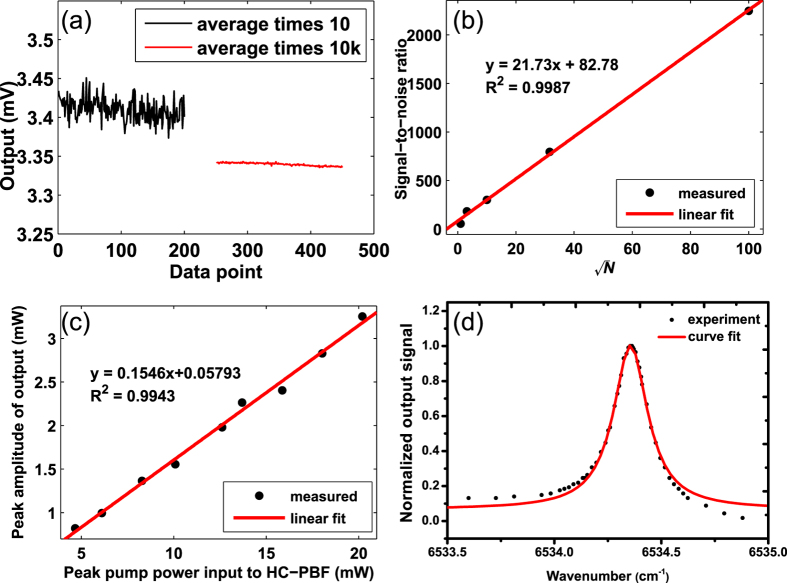
Results of gas detection and spectral line shape measurements. (**a**) Boxcar output with different number of averages, (**b**) SNR of the output as function of averaging times N. (**c**) Output from balanced detector (BD) for different pump peak power delivered to the HC-PBF. (**d**) The spectral shape of absorption line measured with the PTI system.

The performance of the pulsed PTI system for gas detection was evaluated with the same setup as shown in Fig. 5 but the oscilloscope is replaced by a boxcar averager (SRS250). The sensitivity of boxcar averager is 1 V/5 mV and gate width is about 300 ns. The pump pulse duration is set to be 3*μs* with repetition rate of 500 Hz. The peak pump power launched into HC-PBF is 20.2 mW. The *C*_2_*H*_2_ gas concentration is 7500 ppm. Figure 8a shows the output from the Boxcar for the averaging times (*N*) of 10 and 10000. The 1*σ* noise level is reduced significantly with larger number of averages but the signal level remains approximately the same. The slight change (~0.06 dB) in the averaged signal amplitude is believed due to the stability of our phase detection system. The measurements with 10 and 10 K averages were conducted with a time interval of ~20 min, and the stability of our Sagnac demodulation system was previously measured to be ~0.87 dB over a 6-hour period^18^, due to possibly polarization state variations. The relationship between signal-to-noise ratio (SNR) and the number of averaging (*N*) is calculated and shown in Fig. 8b. Curve fitting shows that the SNR is linearly proportional to (*N*)^1/2^, indicating that the noise in our measurement is approximately white^19^. With averaging times of 10000, the signal output from the BD is ~3.34 *mV*and 1*σ* noise level is 0.00147 mV. The SNR is about 2272. The lower detection limit in terms of noise equivalent gas concentration can then be estimated to be is ~3.3 *ppm* for a SNR of unity. For the pump pulse duration of 3 *μ*s, the peak amplitudes of the PT modulation for varying pump power level is also measured and plotted in Fig. 8c. As expected, it follows a linear relationship, indicating that the system performance would be further improved by increasing the peak pump power level.

The lower detection limit could also be improved by increasing the length of the sensing HC-PBF. However, the use of long HC-PBF would result in slow response due to time taking to fill the hollow-core. The response time may be reduced by introducing micro-channels along the HC-PBF or applying pressure differentials^1^. We have recently demonstrated the drilling of multiple low-loss micro-channels along a single HC-PBF with spacing between the micro-channels of a few cm, which would enable gas HC-PBF gas sensors with response time of well below 1 min^3^,^20^.

The spectral shape of absorption line can also be determined by use of the pulsed photothermal interferometry. Figure 8d shows the boxcar output signal when the pump wavelength is tuned from 1530.27 to 1530.47 nm (corresponding to the wavenumber range of 6533.94 *cm*^−1^ ~ 6534.79 *cm*^−1^). The signal was normalized against the maximum PT signal at the line center. By fitting the data to the Lorentz line shape, we obtained a half width half maximum (HWHM) of 0.09757 *cm*^−1^, quite close to that obtained from HITRAN data base (0.0820 *cm*^−1^).

## Discussion

Theoretical and experimental investigations of phase modulation in pulsed photothermal spectroscopy with a HC-PBF filled with *C*_2_*H*_2_ (with *N*_2_ as the buffer gas) are conducted. A simple theoretical model similar to the heat transfer model for a free-space system but with different boundary condition is developed for investigation on the phase modulation dynamics in the HC-PBF system. The numerically computed phase modulation dynamics agree with the measured results obtained experimentally with pump pulse duration from tens of ns to a few *μs*. Some useful conclusions are drawn and further works are suggested. They are summarized as follow:

- For a fixed peak pump power level, the maximum phase modulation increases with pulse duration and approaches a constant after a critical time interval of ~1.2 *μs*. The maximum phase modulation is linearly proportional to the peak pump power, the length of HC-PBF and gas absorption. With the NKT Photonics’ HC-1550-02 fibre, and for a pump laser operating at the peak of the P(9) absorption line, the phase modulating coefficient was determined to be 1.767 × 10^−6^ *rad* · *ppm*^−1^ · *mW*^−1^ · *m*^−1^or 1.517 *rad* · *cm* · *mW*^−1^ · *m*^−1^.
- For pulse duration of the order of 1 *μs*, the thermal conduction is identified as the main mechanism responsible for the phase modulation dynamics. The characteristic time constant for both leading and trailing time behavior with optimal pulse duration was experimentally determined to be around 300 ns, agree well with the calculated value of *t*_*c*2_/4 ~ 275 *ns* and is also affected by the thermal relaxation time *τ* of gas molecules. The pulse duration required to achieved maximum phase modulation (optimal pulse duration) is approximately ~1.2 *μs*, determined by thermal conduction time of *a/v*_*cond*_ ~ 1.1 *μs*.
- Gas detection experiments with a 1530.371 nm pump laser with peak power of 20.2 mW, pulse duration of 3 *μs* and 500 Hz repetition demonstrated a noise equivalent *C*_2_*H*_2_ concentration of ~3.3 ppm with averaging times of 10000. This value would be further reduced by using a higher peak pump power and a larger number of averaging times.
- For a fixed pulse energy, the maximum phase modulation was theoretically evaluated and found to increase with reducing pulse duration. It is theoretically possible to achieve ppb level lower detection limit for *C*_2_*H*_2_ in *N*_2_ with pulse energy of ~100 *nJ*. pulse duration of 10 ns and sensing HC-PBF of ~1 m. However, for very high pulse energies, the temperature change near the core/cladding boundary could be large and the first-kind of boundary conduction might not be accurate anymore and further investigation is needed.
- Under the condition of constant pump power level, smaller spot size (mode field diameter) enhances pump light intensity in the core, which would enhance the PT phase modulation. On the other hand, smaller core size would mean that thermal conduction is fast and the phase modulation could be reduced for a fixed pulse duration because of the fast thermal dissipation. An optimal HC-PBF with appropriate core size should exist to achieve maximum phase modulation.
- We only investigated pulse duration in the range of few tens of ns to *μs*. For much longer (e.g., millisecond) or shorter (e.g., picoseconds) pulse duration, the phase modulation could be affected by other processes and further investigation is needed.

## Additional Information

**How to cite this article**: Lin, Y. *et al*. Pulsed photothermal interferometry for spectroscopic gas detection with hollow-core optical fibre. *Sci. Rep.* **6**, 39410; doi: 10.1038/srep39410 (2016).

**Publisher's note:** Springer Nature remains neutral with regard to jurisdictional claims in published maps and institutional affiliations.

## Supplementary Material

Supplementary Information

## Figures and Tables

**Table 1:** Peak phase modulation for different sizes of the hollow-core.

Core size(*μm*)	Maximum phase changes (rad)
5.5	0.001
9.5	0.0052
20	0.0085
50	0.012

## References

[b1] Jin W., Ho H. L., Cao Y. C., Ju J. & Qi L. F. Gas detection with micro-and nano-engineered optical fibers. Optical Fiber Technology. 19(6), pp.741–759 (2003).

[b2] Russell Philip St. J. Photonic-Crystal Fibers. J. Lightwave Technol. 24, 4729–4749 (2006).

[b3] Hoo Y. L., Liu S., Ho H. L. & Jin W. Fast response microstructured optical fiber methane sensor with multiple side-openings. IEEE Photonics Technology Letters. 22(5), 296–8 (2010).

[b4] Parry J. P. . Towards practical gas sensing with micro-structured fibres. Meas. Sci. Technol. 20(7), 075301 (2009).

[b5] Yang F., Jin W., Cao Y. C., Ho H. L. & Wang Y. P. Towards high sensitivity gas detection with hollow-core photonic bandgap fibers. Opt. Express. 22(20), pp.24894–24907 (2014). 25322061 10.1364/OE.22.024894

[b6] Jin W., Cao Y. C., Yang F. & Ho H. L. Ultra-sensitive all-fibre photothermal spectroscopy with large dynamic range. Nat. Commun. 6, (2015). 10.1038/ncomms7767 PMC4403440 25866015

[b7] Davis C. C. & Petuchowski S. J. Phase fluctuation optical heterodyne spectroscopy of gases. Appl Optics 20, 2539–2554 (1981). 10.1364/AO.20.002539 20332989

[b8] Bialkowski S. Photothermal spectroscopy methods for chemical analysis (John Wiley & Sons, 1996).

[b9] Wang J. C. & Springer G. S. Vibrational relaxation times in some hydrocarbons in the range 300–90 K. The J. Chem. Phys. 59, 6556–6562 (1973)

[b10] Barber R. W. & Emerson D. R. The influence of Knudsen number on the hydrodynamic development length within parallel plate micro-channels. Advances in fluid mechanics. 32, 207–216 (2002).

[b11] Smith D. C. Thermal defocusing of CO_2_ laser radiation in gases. Quantum Electronics. 5, 600–607 (1969).

[b12] Aghaie K. Z., Digonnet M. J. F. & Fan S. H. Experimental Assessment of the Accuracy of an Advanced Photonic-Bandgap-Fiber Model. J. Lightwave Technol. 31, 1015–1022 (2013).

[b13] Davis M. K., Digonnet M. & Pantell R. H. Thermal effects in doped fibers. J. Lightwave Technol. 16, 1013–1023 (1998).

[b14] Snyder A. W. & Love J. Optical waveguide theory. Springer Science & Business Media (2012).

[b15] Monson B., Vyas R. & Gupta R. Pulsed and cw photothermal phase shift spectroscopy in a fluid medium: theory. Appl Optics. 28, 2554–2561 (1989). 10.1364/AO.28.002554 20555557

[b16] Kråkenes K. & Bløtekjaer K. Sagnac interferometer for underwater sound detection: noise properties. Opt Lett. 14, 1152–1154 (1989). 19753085 10.1364/ol.14.001152

[b17] Ma J., Yu Y. Q. & Jin W. Demodulation of diaphragm based acoustic sensor using Sagnac interferometer with stable phase bias. Opt. Express. 23, 29268–29278 (2015) 26561196 10.1364/OE.23.029268

[b18] Lin Y. C., Jin W., Yang F. & Wang C. Highly sensitive and stable all-fiber photothermal spectroscopic gas sensor. In CLEO : Science and Innovations pp. STu4H-3 (2016).

[b19] Werle P., Slemr F., Gehrtz M. & Bräuchle C. Quantum-limited FM-spectroscopy with a lead-salt diode laser. Applied Physics B 49, 99–108 (1989). 10.1364/AO.28.001638 20548718

[b20] Yang F., Tan Y. Z., Jin W., Lin Y. C., Qi Y. & Ho H. L. Hollow-core fiber Fabry–Perot photothermal gas sensor. Opt Lett. 2016 Jul 1, 41(13), 3025–8. 27367092 10.1364/OL.41.003025

