# Supplementary material for: Pulsed photothermal interferometry for spectroscopic gas detection with hollow-core optical fibre
Source: Sci Rep. 2016 Dec 23;6:39410. doi: 10.1038/srep39410 (PMC5180182; doi:10.1038/srep39410)
Supplement: Supplementary Information [file srep39410-s1.pdf]

Supplementary materials:

Pulsed photothermal interferometry for spectroscopic gas detection with hollow-core optical fibre

Yuechuan Lin, Wei Jin, Fan Yang, Jun Ma, Chao Wang,

Hoi Lut Ho, Yang Liu

S1 Output waveform discussion

Fig. S1 illustrates the phase changes of the CW and CCW waves as well as the output from the BD for idealized pulsed phase modulations with duration of $2\ \mu s$ and $15\ \mu s$. The CW and CCW waves experience the same phase modulation generated by the absorption of the pump pulse within gas-filled HC-PBF but with a delay time of $t_d \sim 10\ \mu s$. The output waveform from the BD, which is proportional to the phase difference between the CW and CCW waves, can however be different for different duration of the pump pulses. For pump duration smaller than the loop delay time (i.e., 10 μs), a pair of independent pulses with the same shape but reverse sign appears at the BD output, and the pulse shape is identical to that of the phase modulation in the HC-PBF. However, when the pulse duration is larger than the loop delay time, the output from the BD can be very different from the phase modulation of the individual CW or CCW waves and the pulse duration of output signal is determined by the loop delay time t_d.

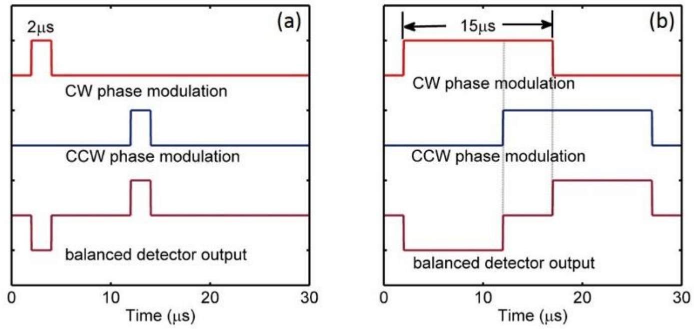

Supplementary Figure S1. Sketches of signals in the Sagnac interferometer. The pump duration of the pump is (a) short ($2\ \mu s$) and (b) long ($15\ \mu s$).

Fig. S2 shows output from the BD recorded with a digital oscilloscope. The oscilloscope has been set to an averaging mode with average times of 128 and a device bandwidth of 20 MHz. As discussed above, the pair of pulses for shorter pump pulse duration (Fig. S1a) are identical but have opposite signs. In our main article, we focus on the pulse with a positive amplitude to study the dynamics of PT phase modulation.

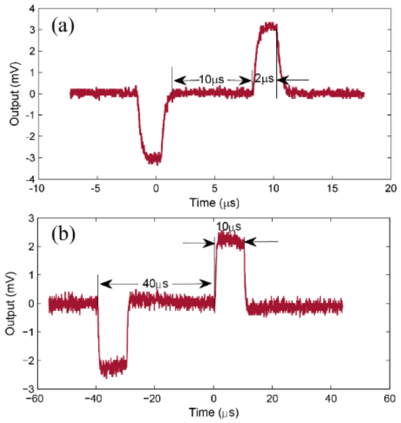

Supplementary Figure S2. Balanced detector outputs. Pump pulse duration is (a) 2 μs and (b) 40 μs
